# Inhibition of Mast Cell Degranulation Relieves Visceral Hypersensitivity Induced by Pancreatic Carcinoma in Mice

**DOI:** 10.1007/s12031-019-01352-6

**Published:** 2019-06-14

**Authors:** Dawei Yu, Jiao Zhu, Mei Zhu, Kai Wei, Qianbo Chen, Xiaodan Wu, Xuerong Miao, Zhijie Lu

**Affiliations:** 1 Department of Anesthesiology and Intensive Care, Third Affiliated Hospital of Second Military Medical University, 225 Changhai Road, No.2 Building, 3rd Floor, Shanghai, 200438 China; 2 Department of Anesthesiology, 101st Hospital of CPLA, Wuxi, Jiangsu China; 3 Department of Anesthesiology, Fujian Provincial Hospital, Fujian Provincial Clinical Medical College, Fujian Medical University, Fuzhou, 350001 Fujian People’s Republic of China

**Keywords:** Pancreatic carcinoma, Cancer pain, Mast cells, Visceral hypersensitivity, Nociception

## Abstract

Cancer pain induced by pancreatic carcinoma is one of the most common symptoms and is difficult to endure, especially in the advanced stage. Evidence suggests that mast cells are recruited and degranulate in enteric disease-related visceral hypersensitivity. However, whether mast cells promote the visceral pain induced by pancreatic carcinoma remains unclear. Here, using toluidine blue staining and western blotting, we observed that mast cells were dramatically recruited to tissues surrounding pancreatic carcinoma, but not inside the carcinoma in patients with severe visceral pain. The levels of mast cell degranulation products, including tryptase, histamine, and nerve growth factor, were significantly increased in pericarcinoma tissues relative to their levels in normal controls, as evidenced by enzyme-linked immunosorbent assay. We determined that systemic administration of mast cell secretagogue compound 48/80 exacerbated pancreatic carcinoma-induced visceral hypersensitivity in a male BALB/c nude mouse model as assessed by measuring the hunching behavior scores and mechanical withdrawal response frequency evoked by von Frey stimulation. In contrast, the mast cell stabilizer ketotifen dose-dependently alleviated pancreatic cancer pain. In addition, we observed incomplete development of abdominal mechanical hyperalgesia and hunching behavior in mast cell–deficient mice with pancreatic carcinoma. However, ketotifen did not further attenuate visceral hypersensitivity in mast cell–deficient mice with carcinoma. Finally, we confirmed that intraplantar injection of pericarcinoma supernatants from BALB/c nude mice but not mast cell–deficient mice caused acute somatic nociception. In conclusion, our findings suggest that mast cells contribute to pancreatic carcinoma-induced visceral hypersensitivity through enrichment and degranulation in pericarcinoma tissues. The inhibition of mast cell degranulation may be a potential strategy for the therapeutic treatment of pancreatic carcinoma-induced chronic visceral pain.

**Electronic supplementary material:**

The online version of this article (10.1007/s12031-019-01352-6) contains supplementary material, which is available to authorized users.

## Introduction

Pancreatic cancer is a kind of malignant carcinoma of the digestive system with poor prognosis. Pain is usually one of the first symptoms in patients with pancreatic cancer. In the advanced stage of pancreatic cancer, patient suffering involves not only physical but also social, psychological, and spiritual aspects. Some patients are more fearful of cancer pain than of death. Therefore, relieving cancer pain has become an indispensable part of pancreatic cancer treatment.

Currently, it is believed that a variety of factors are involved in the generation of pancreatic cancer pain, but the exact mechanisms are unclear. It is considered that pancreatic cancer pain is a kind of distinct pain that manifests as chronic visceral pain and is induced by the progressive growth and invasion of pancreatic tumor cells (Bapat et al. 2011). Previous evidence suggests that mast cells can be recruited by chemokines, including adrenomedullin, C-C chemokine ligand 2, and hepatocyte cytokines and activated by mast cell activators containing corticotrophin-releasing hormone, IL-1, and somatostatin, which are released by tumor cells (Theoharides 2008). Interestingly, a few studies found a potential correlation between the activation of abundant mast cells and visceral pain in irritable bowel syndrome (IBS), cystitis, complex regional pain syndrome, and chronic pancreatitis (Bicer et al. 2015; Demir et al. 2013; Xu et al. 2017). Mast cells located around nerve fibers usually interact with the nerve fibers by releasing mediators such as nerve growth factors (NGFs), histamine, and tryptase (Grundy 2004). Therefore, sensory fibers innervating the visceral organs that transduce and transmit nociceptive signals may be regulated and even activated by the recruited mast cells after activation. Studies have shown that the content of histamine and tryptase in colon biopsy specimens is related to the extent of visceral hyperalgesia in IBS mice (Buhner et al. 2009). In addition, mast cell–deficient mice (MCD) exhibit blunted responses to abdominal mechanical stimulation, making the contribution of mast cells in visceral hyperalgesia more convincing (Hoogerwerf et al. 2005). It was reported that the number of mast cells in the pancreas increased dramatically in patients with painful chronic pancreatitis but not in patients with painless pancreatitis (Hoogerwerf et al. 2005). However, whether and how mast cells are involved in pancreatic cancer pain is unclear.

Studies have shown that mast cells also have a tendency for enrichment and activation in pancreatic cancer. However, there is no clear relationship between pancreatic cancer pain and tumor size and location (Longo et al. 2018). The occurrence and maintenance of pancreatic cancer pain may involve many factors, and dominant factors remain unclear. Currently, the most widely studied pathway pain in visceral pain is the NGF signaling pathway (Matricon et al. 2013). The combination of NGF secreted by mast cells with TrkA and/or p75 NTR on nerve fibers directly activates and sensitizes sensory nerves near pancreatic tumors (Zhang et al. 2018). It was found that NGF regulates the expression and function of transient receptor potential vanilloid 1 (TRPV1, a nonselective cationic channel), which activates the sensory nerves in mice with chronic pancreatitis (Zhu et al. 2011). The activation of TRPV1 results in the depolarization of neurons and the release of calcitonin-related gene peptide (CGRP) and substance P (SP), which send pain signals through the central nervous system (Quartu et al. 2014). Although research on pancreatic cancer pain mechanisms and their clinical treatment methods is progressing, there are still many limitations. For example, it is impossible to replicate the entire process of pancreatic cancer cell development, progression, and metastasis in animal models; the localization of mast cells has not been clarified; and basic research has not been effectively converted into clinical applications. Therefore, research on pancreatic cancer pain has progressed slowly.

In this study, we speculate that mast cells are locally recruited and activated by pancreatic carcinomas and release mediators such as tryptase, histamine, and NGF. To this end, we first observed the distribution of mast cells in pancreatic tumors and determined whether these cells were related to the pain scores. Then, we investigated the key role of mast cells underlying pancreatic cancer pain in mouse models by using mast cell stabilizers and activators, mast cell–deficient mice, etc.

## Materials and Methods

### Patients and Specimens

Pancreatic tumor tissues and peritumor tissues were obtained from 9 patients who had mid-abdominal pain and were scheduled for pancreaticoduodenectomy or body-tail pancreatectomy (6 patients with pancreatic head adenocarcinoma and 3 patients with pancreatic body-tail carcinoma) at Eastern Hepatobiliary Surgery Hospital between January 2015 and January 2016. The 9 control patients were patients with common bile duct cancer, and their pancreatic tissues were used as control specimens in this study. The study was approved by the Ethical Committee of Eastern Hepatobiliary Surgery Hospital, Second Military Medical University. Written informed consent was obtained from all patients when they were recruited. The data do not contain any information that could identify the patients. The diagnosis of pancreatic carcinoma was confirmed by pathology after surgery in all 9 patients. All patients were assessed using a visual analogue scale (VAS) before surgery. Clinicopathological characteristics are summarized in supplemental Tables 1 and 2.

### Animal

Clean healthy male C57/BL6 (20–25 g) and athymic BALB/c nude (20–25 g), a strain with a genetic mutation that causes a deteriorated or absent thymus that increased the success rate of orthotopic pancreatic cancer model without affecting the mast cell, were provided by the animal center of Second Military Medical University, China. Mast cell–deficient *Kit*^*W-sh/W-sh*^ mice on a C57/BL6 genetic background were purchased from The Jackson Laboratory (Bar Harbor, ME). Animals were raised on a 12-h/12-h light/dark cycle in a temperature-controlled room (22–25 °C) with water and food pellets available ad libitum. Group sizes were based on previous experience without a priori statistical power calculation. Mice were randomly assigned to treatment groups. The animal use protocol was approved by the Institutional Animal Care and Use Committee of Second Military Medical University. The procedures were consistent with the ethical guidelines of the National Institutes of Health and the International Association for the Study of Pain. All of the experiments were performed with double-blind methods.

### Histological Evaluation

Specimens of pancreatic tumors, peripancreatic tumor tissues, and normal pancreatic tissues were quickly fixed in a 4% buffered formaldehyde solution. After dehydration, tissues were embedded in paraffin and sectioned at a thickness of 4–5 μm. After dewaxing with xylene, sections were stained with hematoxylin and eosin (H&E) and toluidine blue according to standard methods. Then, sections were sealed with neutral resin and prepared for observation and imaging. Images were acquired using a DXM1200 digital camera (Nikon, Nikon Instruments, Düsseldorf, Germany) attached to an Eclipse E600 optical microscope (Nikon, Nikon Instruments, Düsseldorf, Germany) and imported to the computer. Toluidine blue–stained mast cells were counted in 10 fields/section and the histoarchitectural features were then defined. Researchers performing cell counts remained blinded to the tissue source.

### Enzyme-Linked Immunosorbent Assay

Fresh specimens were cut into small pieces (1 mm^3^), rinsed in saline, and incubated immediately in Hanks’ Balanced Salt Solution (HBSS) (100 mg specimens in 2 ml HBSS) at 37 °C for 25 min. After incubation, the solutions used for histamine determination were quickly heated to 95 °C to prevent degradation by histaminase. All incubated solutions were centrifuged (3000 rpm, 4 °C, 15 min), and supernatants were collected and stored at − 80 °C until further use. For all experiments, supernatant volumes were standardized to the weight of the incubated specimens and not to the supernatant protein, as the protein content was below the detection threshold in the specimen supernatants.

The concentrations of tryptase, histamine, and NGF in human pancreatic carcinoma and normal pancreatic tissues were measured by ELISA using ELISA kits (Shanghai Boyun Bio-Technology Co., Ltd., Shanghai, China) for human tryptase, histamine, and NGF. Each test was performed strictly in accordance with the manufacturer’s instructions.

### Western Blotting

Protein extraction and western blot analysis were carried out as described (Miao et al. 2017). Briefly, lysate was obtained from specimens of pancreatic tumors and peripancreatic tumor tissues. Specimens were ground using a low-speed tissue homogenizer. All operations were performed on ice. Homogenates were centrifuged (12,000 rpm, 15 min, 4 °C), and the supernatant was collected. The protein content was measured with a BCA protein assay. Subsequently, protein was denatured at 99 °C for 5 min. Equal amounts of protein (30 μg/sample) were loaded onto sodium dodecyl sulfate-polyacrylamide gels, electrophoresed, and transferred onto polyvinylidene difluoride membranes. Membranes were incubated overnight at 4 °C with mouse monoclonal anti-β-actin antibody (1:3000, Abcam, ab5694) and rabbit monoclonal anti-mast cell tryptase antibody (1:500, Abcam, ab151757), followed by anti-mouse IgG-horseradish peroxidase (HRP) (1:5000, Cell Signaling Technology, #7076) and anti-rabbit IgG-HRP (1:5000, Cell Signaling Technology, #7074) antibodies, respectively, for 2 h at room temperature. A chemiluminescence reagent kit (ECL, Bio-Rad, Hercules, CA, USA) was used to detect the immunoreactive bands. Protein bands were normalized to those of β-actin. Image-Pro Plus 6.0 software was used to quantitate the protein content.

### The Mouse Model of Pancreatic Cancer

An orthotopic pancreatic cancer model was established by using a human pancreatic cancer cell line (SW1990, American Type Culture Collection, USA) as described previously (Wang et al. 2017). Briefly, BALB/c nude mice and mast cell–deficient mice were anesthetized with sodium pentobarbital (40 mg/kg) and fixed in the supine position. A 1 cm incision was made in the left upper abdomen, and the pancreas was exteriorized. A 20 μl volume of tumor cell solution was injected into the head of the pancreas using a microsyringe. Next, the pancreas was returned to the peritoneal cavity. The abdomen (peritoneum and skin) was closed in layers. In sham-operated mice, boiled SW1990 cells were injected. Nociceptive testing was performed before and on 7 days, 14 days, and 21 days after injection of the SW1990 cells.

### Behavior Tests

#### Abdominal Mechanical Hyperalgesia

Abdominal mechanical hyperalgesia was evaluated using von Frey filaments before and on 7 days, 14 days, and 21 days after the injection of SW1990 cells. Each mouse was placed in a Plexiglas chamber underneath a mesh screen for 30 min. After the mice were quiet, two different filaments (0.02 g and 0.16 g) were used to stimulate the left upper abdomen for approximately 2 s and each stimulation was repeated 10 times at 5 min intervals. A withdrawal response was defined as lifting the belly, scratching, licking the abdomen, or immediate movement or jumping. The occurrence of a withdrawal response in each of these ten trials was expressed as the percent response frequency [(number of withdrawals/10 trials) × 100 = % response frequency], and this percentage was used to indicate of the amount of withdrawal responses.

#### Hunching Score

Hunching behavior was quantified as previously described (Wang et al. 2017). Briefly, mice were placed individually in the center of an open field arena (10 × 10 × 30 cm) with solid black walls and a black floor. Hunching behavior was scored on a scale from 0 to 4: (0) normal, (1) mild hunching, (2) severe hunching, (3) severe hunching with reduced movement, and (4) no movement. In all cases, observations were performed in a blinded manner to the experimental status of the mouse with two independent investigators confirming the results.

#### Heat Nociception

Heat nociception (the Hargreaves test) was assessed using a model IITC 390 analgesic meter (IITC Inc./Life Science Instruments, MI, USA). Each mouse was placed in a Plexiglas chamber underneath a glass table (3 mm) for 30 min. After the mice were quiet, the latency of paw withdrawal was recorded from the start of the radiant heat until the foot lift. Intensity of light radiation was set to make the baseline paw withdrawal latency at 10 ± 2 s. Based on our experiments, an intensity of 18% was used for the evaluation of heat nociception. The radiation was automatically stopped at 20 s to avoid damage to the hind paw. Each trial comprised 5 tests at 10 min intervals, and the average was taken.

#### Walking Score

The walking score was the indicator of exercise-induced pain. Briefly, each mouse was placed in a Plexiglas chamber with a mesh screen on the bottom. The use of the left hind limb during walking was evaluated: (0) normal use, (1) slight limp, (2) severe limp, and (3) complete lack of limb use.

Mechanical nociception, heat nociception, and walking scores were evaluated at 1 h, 2 h, 4 h, and 8 h after injection of the supernatants of peripancreatic carcinoma tissues or vehicle.

#### Open-Field Test

To evaluate the sedative effect of ketotifen, the open-field (OF) test was performed. The nude mice received a single intraperitoneal injection of ketotifen or vehicle, and spontaneous locomotor activity was assessed using the open-field test 1 h after the injection. This behavioral test was assessed in an open field (20 cm × 20 cm × 20 cm. Shanghai Jiliang Software Technology Co. Ltd., Shanghai, China) for 10 min. The total distance and time traveled in the field was recorded as a measure of spontaneous exploratory activity.

### Statistical Analyses

The animals were randomly distributed into various treatment groups in this study. The data were analyzed using GraphPad Prism 5 software. All animal experiment results are presented as the means ± SEMs. Statistical significance was set at *P* < 0.05. The results from the behavioral tests, western blotting, and staining were statistically analyzed with one-way or two-way analysis of variance (ANOVA). When ANOVA showed a significant difference, pairwise comparisons between means were tested by the post hoc Bonferroni method. The Mann-Whitney *U* test was used to analyze age, VAS, and ELISA data. The chi-square test was used to analyze sex data of clinic patients.

## Results

### Mast Cells Were Recruited to Tissues Surrounding Pancreatic Tumors in Patients with Severe Visceral Pain

To explore the role of mast cells in pancreatic cancer-induced visceral hyperalgesia, we first examined the mast cell distribution pattern in pancreatic cancer and adjacent tissues in human surgical specimens. Nine surgical specimens of human pancreatic cancer tissues and 9 surgical specimens of normal pancreatic tissues were obtained (Table 1). Notably, the patients who donated the cancer tissues were all those with visual analogue scale (VAS) scores of ≥ 5 and the patients who donated normal pancreatic tissues were all those with VAS = 0. There were no significant differences in age and sex between the two groups.

**Table 1:** Clinical data of human specimens

Characteristic	Control (*n* = 9)	Pancreatic carcinoma (*n* = 9)
Age (year, range)	26–62	48–61
Sex (no., F/M)	2/7	3/6
VAS (mean ± SD)	0	6.1 ± 1.3**

**P < 0.01 compared with control group

**Fig. 1:**
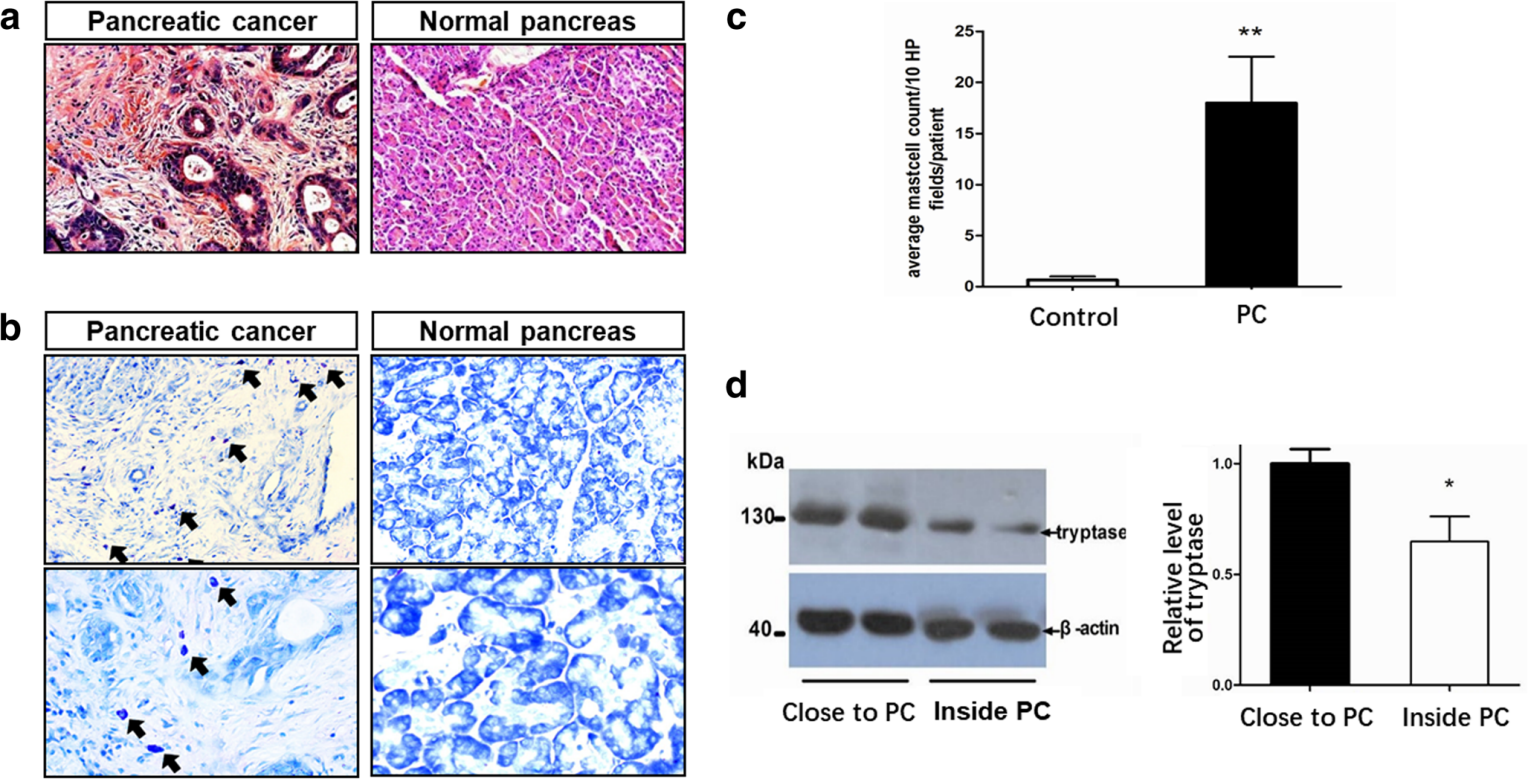
The enrichment of mast cells surrounding pancreatic carcinoma in human specimens *n* = 9 specimens/group. **a** H&E staining of pancreatic carcinoma (left) and normal pancreatic tissues (right). **b** Toluidine blue/H&E staining of pancreatic carcinoma (left) and normal pancreatic (right) tissues at × 200 (upper) and × 400 (lower) magnification. Mast cells were predominantly localized in the tissues surrounding the pancreatic carcinoma. **c** Statistical summary of the numbers of toluidine blue–stained mast cells in the tissues surrounding the pancreatic carcinoma and in normal pancreatic tissues. **d** The expression of tryptase in the tissues near and inside the pancreatic carcinoma. *n* = 4 specimens. One-way ANOVA, *P* < 0.05. PC, pancreatic carcinoma. *P < 0.05 or **P < 0.01

H&E staining showed destroyed pancreatic structures in pancreatic cancer tissues and poorly differentiated pancreatic cells surrounding the cancer tissues (Fig. 1a). To observe the mast cells clearly, we used toluidine blue staining and found a significant enrichment of mast cells surrounding the pancreatic tumors but not in the tumors (Fig. 1b). Statistical analysis revealed a significantly increased amount of mast cells in the pericarcinoma tissues compared with that in normal pancreatic tissues (Fig. 1c, 18.00 ± 4.52 vs 0.67 ± 0.33, respectively, one-way ANOVA, *P* < 0.05). Under high magnification, some of the recruited mast cells surrounding the carcinoma showed degranulation in histological sections.

To quantify mast cell recruitment in pancreatic tumor tissues and pericarcinoma tissues, tryptase, a proteolytic enzyme existing predominantly in mast cells, was used as a marker of mast cells for western blotting. The expression of tryptase was significantly elevated in pericarcinoma tissues compared to that in tumor tissues (Fig. 1d). These data provide evidence that mast cells are predominantly recruited and activated in the pericarcinoma tissues of patients with severe visceral pain. Therefore, in the next experiments, pericarcinoma tissues, not pancreatic carcinoma tissues, were selected for study.

### Increased Amounts of Mast Cell Degranulation Products Pericarcinoma Tissues: Histamine, Tryptase, and NGF

**Fig. 2:**
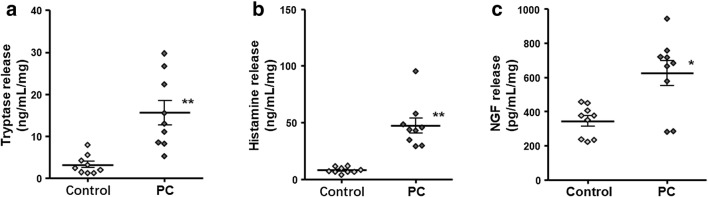
The release of tryptase, histamine, and NGF in pericarcinoma tissues and normal pancreatic tissues (control), as detected by ELISA. **a** The expression of tryptase was increased in pericarcinoma tissues compared to that in control tissues. *n* = 3 replicates (9 specimens)/group. Two-tailed Student’s *t* test, *P* = 0.0002. **b** The expression of histamine was increased in pericarcinoma tissues compared to that in control tissues. *n* = 3 replicates (9 specimens)/group. Two-tailed Student’s *t* test, *P* < 0.0001. **c** The expression of NGF was increased in pericarcinoma tissues compared to control that in tissues. *n* = 3 replicates (9 specimens)/group. Two-tailed Student’s *t* test, *P* = 0.0134. *P < 0.05 or **P < 0.01

To further investigate the mast cell degranulation products in tissues surrounding the pancreatic tumors, we examined whether the expression of histamine, tryptase, and NGF was altered in the specimens. Fresh surgical specimens were washed and used for ELISA. The results indicated that the expression level of tryptase in pericarcinoma tissues was approximately 4.9-fold greater than that in normal pancreatic tissues (Fig. 2a; 3.17 ± 0.76 ng/ml/mg vs 47.46 ± 6.73 ng/ml/mg, respectively; two-tailed Student’s *t* test, *P* < 0.01). Consistent with this finding, the expression levels of histamine and NGF in pericarcinoma tissues were increased by 5.9-fold (8.05 ± 0.88 ng/ml/mg vs 47.46 ± 6.73 ng/ml/mg, respectively; two-tailed Student’s *t* test, *P* < 0.01) and 1.8-fold (344.0 ± 30.7 pg/ml/mg vs 623.8 ± 72.1 pg/ml/mg, respectively; two-tailed Student’s *t* test, *P* < 0.05), respectively, compared to those in the normal pancreatic tissues (Fig. 2b and c). These results indicate that the amount of mast cell degranulation products was increased in pericarcinoma tissues and further confirmed that mast cells were recruited, infiltrated, and activated in tissues surrounding the pancreatic tumors.

### Activation of Mast Cells Exacerbated Nociception Induced by Pancreatic Cancer

**Fig. 3:**
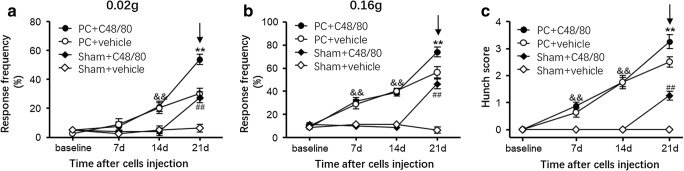
Effect of systemic administration of compound 48/80 on pancreatic carcinoma-induced visceral nociceptive hypersensitivity and spontaneous pain. **a** The withdrawal response frequency to 0.02 g von Frey filament stimulation was increased significantly in pancreatic carcinoma model mice and sham BALB/c nude mice after compound 48/80 injection on day 21 (two-way ANOVA (treatment × time) followed by Bonferroni post hoc tests. *F*_treatment_(3127) = 25.75, *P* < 0.0001. *F*_interaction_(9127) = 10.33, *P* < 0.0001. *n* = 8 mice/group). **b** The withdrawal response frequency to 0.16 g von Frey filament stimulation was increased significantly in pancreatic carcinoma model mice and sham BALB/c nude mice after compound 48/80 injection on day 21 (two-way ANOVA (treatment × time) followed by Bonferroni post hoc tests. *F*_treatment_(3127) = 77.54, *P* < 0.0001. *F*_interaction_(9127) = 19.48, *P* < 0.0001. *n* = 8 mice/group). **c** Hunching score in pancreatic carcinoma model mice and sham BALB/c nude mice after compound 48/80 injection (two-way ANOVA (treatment × time) followed by Bonferroni post hoc tests. *F*_treatment_(3127) = 120.7, *P* < 0.0001. *F*_interaction_(9127) = 25.79, *P* < 0.0001. *n* = 8 mice/group). **P < 0.01 vs vehicle-treated pancreatic carcinoma model mice, ^##^*P* < 0.01 vs vehicle-treated sham BALB/c nude mice. ^&&^*P* < 0.01 vs sham BALB/c nude mice. The arrows indicate the time of vehicle/compound 48/80 administration

To define the role of mast cells in pancreatic cancer pain, we established an orthotopic pancreatic cancer model in BALB/c nude mice (Wang et al. 2017). The BALB/c nude mouse model exhibits hunching behavior and abdominal mechanical hyperalgesia, which mimics the visceral pain symptoms induced by pancreatic cancer seen in the clinic to a great extent (Kelsen et al. 1997; Wang et al. 2017). Compound 48/80, a mast cell stimulator, was systemically administered by intraperitoneal injection on day 21 after SW1990 cell injection. Hunching behavior and abdominal mechanical hyperalgesia elicited by 0.02 g and 0.16 g von Frey filaments were evaluated 30 min after the injection of compound 48/80. A withdrawal response was defined as lifting the belly, scratching, licking the abdomen, or immediate movement or jumping. Injection of SW1990 cells dramatically increased mechanical nociceptive responses in BALB/c nude mice on day 7 and 14 (Fig. 3a and b). The administration of compound 48/80 at 2 mg/kg significantly increased the response frequency to 0.02 g von Frey filament stimulation from 30 to 53.5% in tumor-bearing mice on day 21 (Fig. 3a; *n* = 8, *P* < 0.01). The response frequency to 0.16 g von Frey filament stimulation was also dramatically increased in tumor-bearing mice after injection of compound 48/80 (Fig. 3b; *n* = 8; 56.25% vs 73.75%, respectively; *P* < 0.01). Moreover, compound 48/80 at a dose of 2 mg/kg evoked significant abdominal mechanical hyperalgesia in sham BALB/c nude mice. Similarly, 2 mg/kg compound 48/80 significantly increased the hunching scores in both pancreatic carcinoma model mice and sham mice (Fig. 3c), suggesting that mast cell activation can evoke visceral nociceptive hypersensitivity and exacerbated abdominal mechanical hyperalgesia in mice with pancreatic carcinoma. However, whether mast cells contribute specifically to pancreatic cancer pain has not been defined.

### Stabilization of Mast Cells by Ketotifen Dose-Dependently Alleviated Nociception Induced by Pancreatic Cancer

**Fig. 4:**
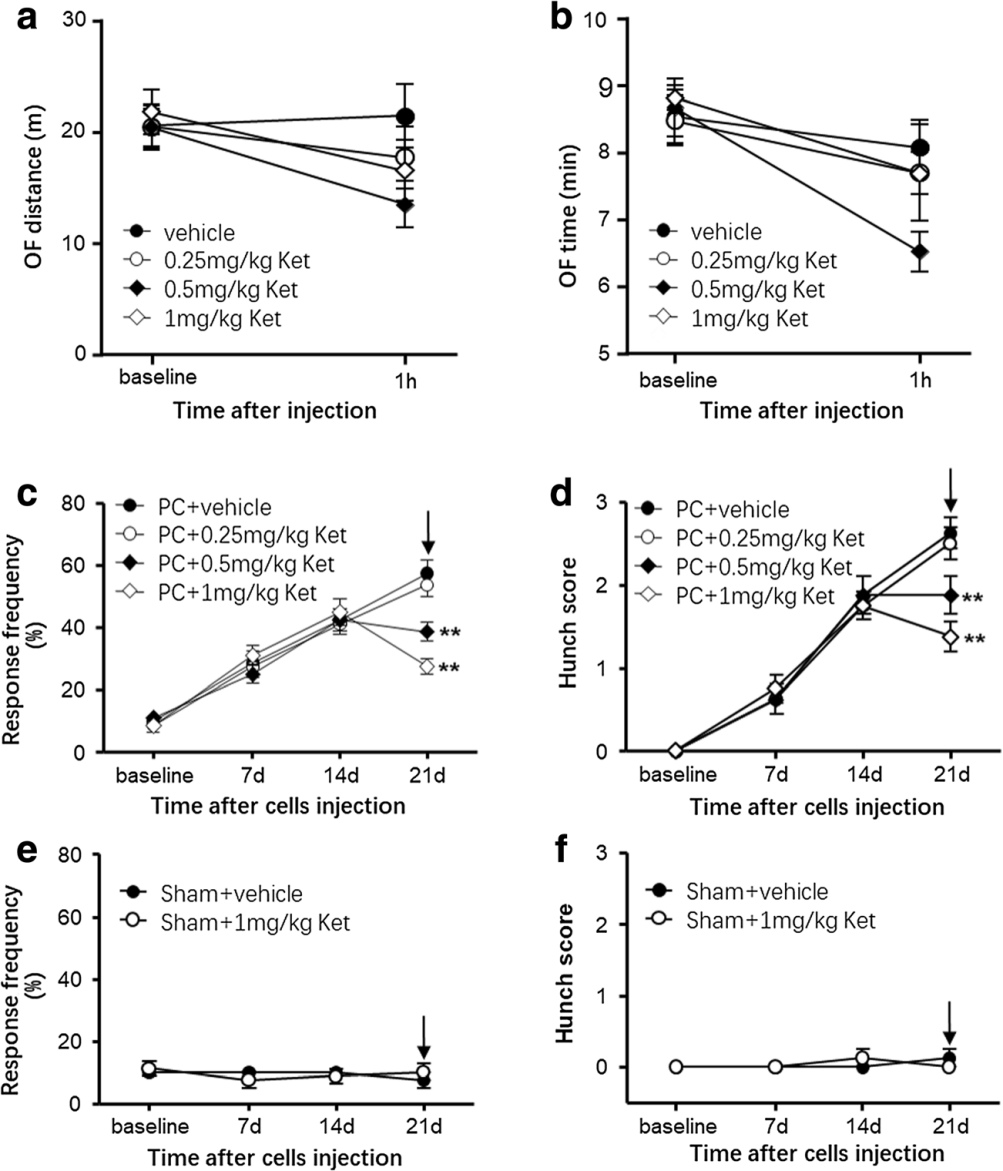
The mast cell stabilizer ketotifen dose-dependently alleviated pancreatic carcinoma-induced visceral nociceptive hypersensitivity and spontaneous pain. **a**, **b** Ketotifen dose-dependently did not have a sedative effect on BALB/c nude mice (**a**, two-way ANOVA (treatment × time). *F*_treatment_ (3.63) = 1.031, *P* = 0.3858. *F*_interaction_ (3.63) = 1.093, *P* = 0.3596. *n* = 8 mice/group. **b**, two-way ANOVA (treatment × time). *F*_treatment_(3.63) = 1.204, *P* = 0.3166. *F*_interaction_ (3.63) = 1.543, *P* = 0.2136. *n* = 8 mice/group). **c** Ketotifen dose-dependently decreased the withdrawal response frequency to 0.16 g von Frey filament stimulation in pancreatic carcinoma model mice (two-way ANOVA (treatment × time) followed by Bonferroni post hoc tests. *F*_treatment_(3127) = 3.676, *P* = 0.0143. *F*_interaction_(9127) = 5.544, *P* < 0.0001. *n* = 8 mice/group). **d** Ketotifen dose-dependently decreased the hunching score in pancreatic carcinoma model mice (two-way ANOVA (treatment × time) followed by Bonferroni post hoc tests. *F*_treatment_(3127) = 2.810, *P* = 0.0428. *F*_interaction_(9127) = 3.286, *P* = 0.0014. *n* = 8 mice/group). **e**, **f** Ketotifen had no effect on the withdrawal response frequency to abdominal mechanical stimulation (**c**, two-way ANOVA (treatment × time). *F*_treatment_(1.63) = 0.000, *P* = 1.000. *F*_interaction_(3.63) = 0.5128, *P* = 0.6751. *n* = 8 mice/group) and the hunching score (**d**, two-way ANOVA (treatment × time), *F*_treatment_(1.63) = 0.000, *P* = 1.000. *F*_interaction_(3.63) = 1.3333, *P* = 0.2727. *n* = 8 mice/group) in sham BALB/c nude mice. **P < 0.01 vs the corresponding vehicle-treated mice. The arrows indicate the time of vehicle/ketotifen administration

To identify the specific role of mast cells in pancreatic cancer pain, we used a mast cell stabilizer, ketotifen, to inhibit the degranulation of mast cells in a pancreatic cancer model and then determined whether the visceral nociceptive hypersensitivity was alleviated. Firstly, we verified whether ketotifen has a sedative effect on BALB/c nude mice by using OF. OF was evaluated 1 h after the injection of ketotifen. The total distance and time traveled in the field was recorded. The results indicated that there were no significant differences in distance and time (Fig. 4a and b, *P* > 0.05) between vehicle and ketotifen (0.25 mg/kg, 0.5 mg/kg, 1 mg/kg) groups. That is, ketotifen did not sedate the BALB/c nude mice. Then, ketotifen or vehicle was administered by intraperitoneal injection to BALB/c nude mice on day 21 post tumor cell inoculation. Ketotifen at 1 mg/kg significantly alleviated pancreatic carcinoma-induced abdominal mechanical hyperalgesia and hunching behavior on day 21 post tumor cell inoculation (Fig. 4c and d). The effects of ketotifen were dose-dependent. On day 21 after tumor cell injection, the 1 mg/kg dose of ketotifen decreased the withdrawal response frequency to 0.16 g von Frey filament stimulation to 48% (*P* < 0.01) and the 0.5 mg/kg dose to 67% (*P* < 0.01) that of the corresponding tumor-bearing mice treated with vehicle. The 0.25 mg/kg dose of ketotifen had no effect on the withdrawal response frequency to mechanical stimulation on day 21 post tumor cell injection. Similarly, the 1 mg/kg dose of ketotifen decreased the hunching score to 52% (*P* < 0.01); the 0.5 mg/kg dose, to 71% (*P* < 0.01); and the 0.25 mg/kg dose, to 95% (*P* > 0.05) that of the corresponding tumor-bearing mice treated with vehicle. As expected, ketotifen did not change the withdrawal response frequency to abdominal mechanical stimulation or the hunching score in sham BALB/c nude mice on day 21 (Fig. 4e and f).

### Incomplete Development of Abdominal Mechanical Hyperalgesia in Mast Cell–Deficient Mice with Pancreatic Cancer

**Fig. 5:**
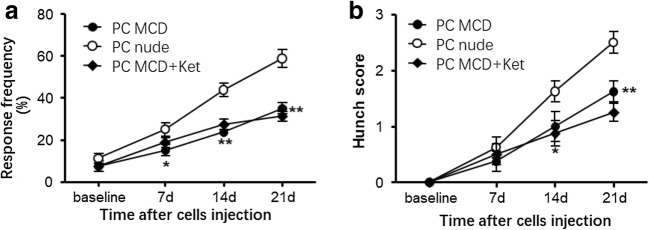
Visceral nociceptive hypersensitivity and spontaneous pain were decreased in mast cell–deficient mice after SW1990 cell inoculation. **a** Mast cell–deficient mice showed a decreased response frequency to mechanical stimulation after SW1990 cell inoculation, and intraperitoneal injection of ketotifen (1 mg/kg) failed to reduce the response frequency further (two-way ANOVA (treatment × time) followed by Bonferroni post hoc tests. *F*_treatment_(2.95) = 34.95, *P* < 0.0001. *F*_interaction_(6.95) = 4.813, *P* = 0.0003. *n* = 8 mice/group). **b** Mast cell–deficient mice showed decreased hunching scores after SW1990 cell inoculation, and intraperitoneal injection of ketotifen (1 mg/kg) failed to reduce the hunching scores further (two-way ANOVA (treatment × time) followed by Bonferroni post hoc tests. *F*_treatment_(2.95) = 10.87, *P* < 0.0001. *F*_interaction_(6.95) = 3.008, *P* = 0.0103. *n* = 8 mice/group). *P < 0.05 or **P < 0.01 vs the corresponding BALB/c nude mice

Nonspecific pharmacological effects of the mast cell activator and stabilizer cannot be excluded from our observations above. To further confirm the role of mast cells in pancreatic pain, we established a pancreatic cancer model in mast cell–deficient mice and observed hunch behaviors and withdrawal response to von Frey filament stimulation. The response to abdominal mechanical stimulation was significantly lower in the mast cell–deficient mice than in BALB/c nude mice from day 7 to 21 after SW1990 cell injection (Fig. 5a, *P* < 0.05). There were no significant differences in the response frequency to abdominal mechanical stimulation with 0.16 g von Frey filaments under baseline conditions between the groups. Consistent with these findings, on days 14 and 21 post SW1990 cell injection, mast cell–deficient mice showed reduced hunching behavior, with significantly decreased scores (Fig. 5b, *P* < 0.05) compared with those of BALB/c nude mice. There was no evidence of hunching behavior in either mast cell–deficient or BALB/c nude mice that received a sham injection. These results showed incomplete development of abdominal mechanical hyperalgesia and spontaneous pain in mast cell–deficient mice after SW1990 cell injection, strongly suggesting the expected role of mast cells in the generation and maintenance of pancreatic cancer pain.

### Supernatants of Pericarcinoma Tissues from BALB/c Nude Mice but Not Mast Cell–Deficient Mice Caused Somatic Nociception

**Fig. 6:**
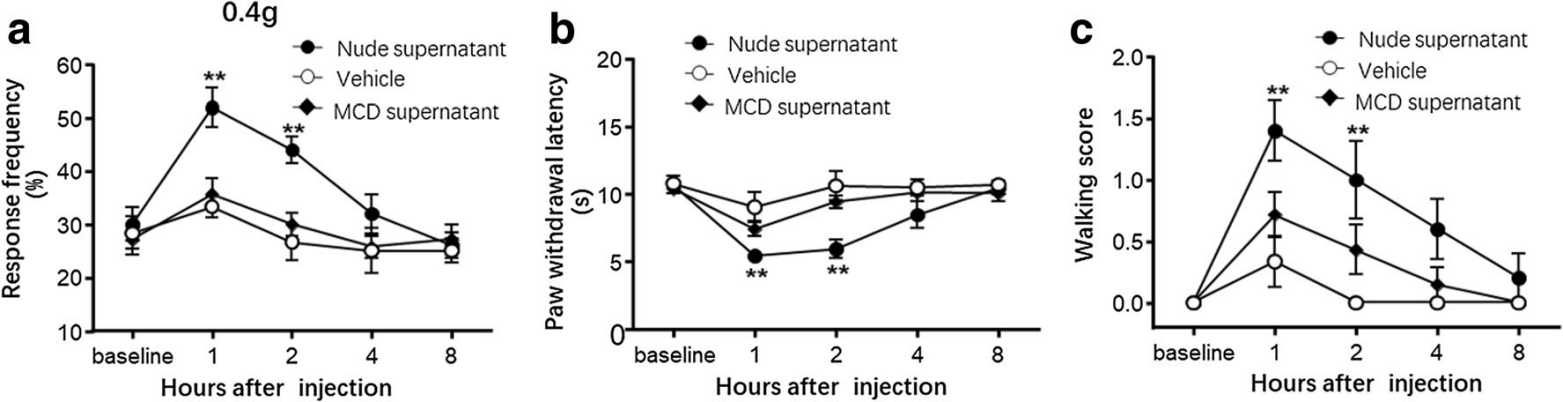
Intraplantar injection from supernatants of pericarcinoma tissues from BALB/c nude mice but not mast cell–deficient mice induced somatic nociception. **a** The withdrawal response frequency to 0.4 g von Frey filament stimulation dramatically increased in mice that received an intraplantar injection of the supernatant pericarcinoma tissues from BALB/c nude mice but not mast cell–deficient mice (two-way ANOVA (treatment × time) followed by Bonferroni post hoc tests. *F*_treatment_(2.89) = 12.48, *P* < 0.0001. *F*_interaction_(8.89) = 2.236, *P* = 0.337). **b** The PWL to thermal stimulation decreased significantly in mice that received an intraplantar injection of the supernatant from pericarcinoma tissues from BALB/c nude mice but not mast cell–deficient mice (two-way ANOVA (treatment × time) followed by Bonferroni post hoc tests. *F*_treatment_(2.89) = 11.40, *P* < 0.0001. *F*_interaction_(8.89) = 2.374, *P* = 0.0246). **c** The walking score decreased significantly in mice that received an intraplantar injection of the supernatant pericarcinoma tissues from BALB/c nude mice but not mast cell–deficient mice (two-way ANOVA (treatment × time) followed by Bonferroni post hoc tests. *F*_treatment_(2.89) = 16.33, *P* < 0.0001. *F*_interaction_(8.89) = 2.216, *P* = 0.0353). *n*_MCD_ = 7 mice, *n*_nude_ = 5 mice, *n*_vehicle_ = 6 mice. **P < 0.01 vs the corresponding mice injected with supernatant from BALB/c nude mice. MCD, mast cell–deficient mice

Finally, we examined the differences in visceral pain between mast cell–deficient mice and BALB/c nude mice after SW1990 cell injection due to pericarcinoma mast cell recruitment. To this end, pericarcinoma tissues from mice models were harvested on day 21 and incubated immediately in HBSS. The supernatants were then standardized and used for the intraplantar injection of naïve mice. After the injection of 20 μl of supernatant from BALB/c nude mice with pancreatic carcinoma, the withdrawal response frequency to 0.4 g von Frey filament stimulation dramatically increased (Fig. 6a, *P* < 0.05), and the paw withdrawal latency (PWL) to thermal stimulation decreased significantly from 1 to 8 h and peaked at 1 h post injection (Fig. 6b, *P* < 0.05). In contrast, there were no significant differences in the response frequency of mechanical stimuli and the PWL of mice injected with supernatant from mast cell–deficient pericarcinoma tissues from 1 to 8 h post injection compared with that of mice injected with HBSS (*P* > 0.05). Moreover, we observed that 8 h after the injection of supernatant from BALB/c nude mice with pancreatic tumors, the increased response frequency of mechanical stimuli and decreased PWLs to thermal stimulation almost completely returned to the baseline values, demonstrating that intraplantar injection of the supernatant induced acute somatic nociception.

Similarly, mice injected with supernatant from BALB/c nude mice showed dramatic increases in walking scores from 1 to 2 h after injection compared to those of mice injected with HBSS (Fig. 6c, *P* < 0.05). However, supernatants of pericarcinoma tissues from mast cell–deficient mice failed to induce significant increases in walking scores after injection compared with the scores of the control group. The results that supernatants from BALB/c nude mice but not mast cell–deficient mice evoked significant acute peripheral nociception suggest that mast cells contribute to visceral hyperalgesia in pancreatic cancer.

## Discussion

In this study, pancreatic carcinoma caused by orthotopic inoculation of SW1990 cells leads to long-term spontaneous nociception visualized as hunching behavior and epigastric mechanical hyperalgesia in a mouse model, which mimics progressive visceral pain in pancreatic carcinoma patients. Understanding the mechanisms that underlie orthotopic carcinoma cell inoculation-induced visceral hypersensitivity may aid the development of a new therapeutic strategy for the prevention and/or treatment of pancreatic cancer pain. Recent studies have shown that the genesis and development of visceral pain may be related to the increase in local nerve fibers accompanied by the growth of pancreatic carcinoma (Ceyhan et al. 2009). However, the local microenvironment, which interacts with nerve fibers, has been an increasing focus on the investigation of peripheral mechanisms of pancreatic cancer pain (Nigri et al. 2017). Here, we report that mast cell degranulation is required for the genesis and maintenance of pancreatic carcinoma-induced visceral hypersensitivity.

The present study demonstrated that mast cells are dramatically recruited to the area of pancreatic carcinoma in patients with abdominal pain. In addition, mast cells predominantly localize close to but not inside the pancreatic carcinoma. Tryptase, a mediator secreted by mast cells, was detected mostly in tissues surrounding the carcinoma. Together with the evidence that increased nerve fibers are found surrounding the carcinoma in the pancreas (Stopczynski et al. 2014), our evidence leads us to believe that mast cells and their degranulation products, including tryptase, histamine, and NGF, may interact with peripheral fibers and ultimately contribute to nociceptive information transduction through direct or indirect pathways.

The recruited mast cells are required for visceral hypersensitivity in mice with pancreatic carcinoma. Our data showed incomplete development of chronic visceral hypersensitivity and the spontaneous nociceptive response in mast cell–deficient mice after inoculation of SW1990 cells. We further confirmed through a pharmacological approach that the mast cell–mediated pancreatic pain mechanism depended on degranulation. A mast cell secretagogue, compound 48/80, was administered systemically to mice in the maintenance stage of chronic pancreatic pain. Enhanced hunching behavior and abdominal mechanical hyperalgesia were observed in mice inoculated with SW1990 cells, demonstrating that mast cell degranulation promotes the development of visceral hypersensitivity in mice with pancreatic carcinoma. However, after intraperitoneal injection of compound 48/80, mice inoculated with boiled SW1990 cells also showed significant hunching behavior and abdominal mechanical hyperalgesia, suggesting that visceral hypersensitivity—not limited to pancreatic pain but also including enteric pain—could be induced by mast cell degranulation alone. Previous reports revealed the crucial role of mast cells in IBS-induced visceral hypersensitivity (Braak et al. 2012; Wang et al. 2014). Compound 48/80 was used as a mast cell stimulator and exacerbated nociception in both pancreatic cancer and sham nude mice here. However, compound 48/80 is also known as an inhibitor of PLC (phospholipase C). Activation of PLC promotes the sensitization of thermoTRPs like TRPA1 and TRPV1 ion channels which were involved in visceral hyperalgesia (Balázs and Bíró 2013). Therefore, from this sight, compound 48/80 was expected to inhibit PLC and repress the nociceptive pathway.

The experiment with compound 48/80 failed to identify whether the exacerbated visceral pain in the pancreatic carcinoma model mice was attributed to the specific role of recruited mast cells in the pancreas or the nonspecific role of widespread intraperitoneal mast cells. Therefore, we carried out a suppression approach by utilizing the mast cell stabilizer ketotifen to inhibit degranulation and evaluated the effect of this treatment on pancreatic cancer pain. Expectedly, intraperitoneal administration of ketotifen effectively inhibited the maintenance of hunching behavior and abdominal mechanical hyperalgesia in a dose-dependent manner, strongly suggesting a specific role of mast cell degranulation in pancreatic cancer pain. Ketotifen did not affect the behaviors of visceral hypersensitivity in the sham group, possibly due to the low quantity and degranulation level of mast cells under normal conditions. Moreover, ketotifen did not restrain hunching behavior or abdominal mechanical hyperalgesia in mast cell–deficient mice with pancreatic carcinoma, indicating that mast cell degranulation contributes partly to the development and maintenance of pancreatic carcinoma-induced visceral hypersensitivity. However, ketotifen has an additional histamine H1 receptor antagonistic effect that might have also contributed to the revealed antinociceptive actions of the agent.

The mast cells involved in pancreatic carcinoma-induced visceral hypersensitivity are likely predominantly localized in tissues surrounding the carcinoma, not inside the carcinoma. Specimens from human pancreatic carcinomas showed a significant increase in tryptase expression in tissues close to the carcinomas compared with that in tissues inside the carcinomas. Then, we confirmed our speculation through a somatic nociception evocation experiment. Intraplantar injection of supernatants from pericarcinoma tissues of BALB/c nude mice but not mast cell–deficient mice induced a 2-h nociceptive response in C57/BL6 mice, which was restored to baseline at 8 h after injection. Our findings demonstrated that mediators from pericarcinoma tissues of BALB/c nude mice, most likely those released during mast cell degranulation, cause somatic nociception and probably play an essential role in the development and maintenance of pancreatic cancer pain.

In human specimens, the levels of mast cell degranulation products, including tryptase, histamine, and NGF, were found to be significantly increased in tissues surrounding pancreatic carcinoma relative to those in normal pancreatic tissues (Grundy 2004). In tumors, these ligands or proteins can produce nociceptive stimuli by activating the corresponding receptors. TRPV1 is one of the key receptors mediating the development of pain sensitivity and is also a necessary condition for the occurrence of cancer pain (Gu et al. 2018; Lapointe et al. 2015). Studies have shown that TRPV1 is coexpressed with protease-activated receptor 2 (PAR2) on dorsal root ganglion neurons. When tryptase binds to PAR2, it activates the TRPV1 receptor, leading to the release of substances such as CGRP and SP, which mediate the occurrence and maintenance of pain (Zhang et al. 2011). In addition, high concentrations of histamine induce an increase in the level of [Ca2+]i by activating PLCβ3, which can promote the release of IP3 (inositol 1,4,5-trisphosphate), eventually leading to the release of calcium from intracellular stores (Imamachi et al. 2009). In addition, histamine binding to H1 receptors can activate TRPV1 channels in sensory neurons through the PLA2/LO (phospholipase A2/lipoxygenase) pathway (Gao et al. 2016). Most CGRP-positive neurons express TrkA and/or p75, which are high- and low-affinity receptors for NGF, respectively (Prato et al. 2017). Therefore, NGF can directly act on these two receptors on visceral primary afferent nerve endings, thus playing a key role in the pathogenesis of pancreatic cancer pain.

In summary, mast cells contribute to pancreatic carcinoma-induced visceral hypersensitivity through enrichment and degranulation in tissues surrounding carcinoma. The inhibition of mast cell degranulation may be a potential strategy for the therapeutic treatment of pancreatic carcinoma-induced chronic visceral pain.

## Electronic Supplementary Material

ESM 1 (DOCX 17 kb)

## References

[CR1] Balázs T, Bíró T (2013). TRP channels and pruritus. The Open Pain Journal.

[CR2] Bapat AA, Hostetter G, Von Hoff DD, Han H (2011). Perineural invasion and associated pain in pancreatic cancer. Nat Rev Cancer.

[CR3] Bicer F, Altuntas CZ, Izgi K, Ozer A, Kavran M, Tuohy VK, Daneshgari F (2015). Chronic pelvic allodynia is mediated by CCL2 through mast cells in an experimental autoimmune cystitis model. Am J Physiol Ren Physiol.

[CR4] Braak B, Klooker TK, Wouters MM, Welting O, van der Loos CM, Stanisor OI, van Diest S, van den Wijngaard RM, Boeckxstaens GE (2012). Mucosal immune cell numbers and visceral sensitivity in patients with irritable bowel syndrome: is there any relationship?. Am J Gastroenterol.

[CR5] Buhner S, Li Q, Vignali S, Barbara G, de Giorgio R, Stanghellini V, Cremon C, Zeller F, Langer R, Daniel H, Michel K, Schemann M (2009). Activation of human enteric neurons by supernatants of colonic biopsy specimens from patients with irritable bowel syndrome. Gastroenterology..

[CR6] Ceyhan GO, Demir IE, Rauch U, Bergmann F, Müller MW, Büchler MW, Friess H, Schäfer KH (2009). Pancreatic neuropathy results in “neural remodeling” and altered pancreatic innervation in chronic pancreatitis and pancreatic cancer. Am J Gastroenterol.

[CR7] Demir IE, Schorn S, Schremmer-Danninger E, Wang K, Kehl T, Giese NA, Algül H, Friess H, Ceyhan GO (2013). Perineural mast cells are specifically enriched in pancreatic neuritis and neuropathic pain in pancreatic cancer and chronic pancreatitis. PLoS One.

[CR8] Gao W, Zan Y, Wang ZJ, Hu XY, Huang F (2016). Quercetin ameliorates paclitaxel-induced neuropathic pain by stabilizing mast cells, and subsequently blocking PKCepsilon-dependent activation of TRPV1. Acta Pharmacol Sin.

[CR9] Grundy D (2004). What activates visceral afferents?. Gut..

[CR10] Gu Y, Li G, Mae Huang LY (2018). Inflammation induces Epac-protein kinase C alpha and epsilon signaling in TRPV1-mediated hyperalgesia. Pain..

[CR11] Hoogerwerf WA, Gondesen K, Xiao SY, Winston JH, Willis WD, Pasricha PJ (2005). The role of mast cells in the pathogenesis of pain in chronic pancreatitis. BMC Gastroenterol.

[CR12] Imamachi N, Park GH, Lee H, Anderson DJ, Simon MI, Basbaum AI, Han SK (2009). TRPV1-expressing primary afferents generate behavioral responses to pruritogens via multiple mechanisms. Proc Natl Acad Sci U S A.

[CR13] Kelsen DP, Portenoy R, Thaler H, Tao Y, Brennan M (1997). Pain as a predictor of outcome in patients with operable pancreatic carcinoma. Surgery..

[CR14] Lapointe TK, Basso L, Iftinca MC, Flynn R, Chapman K, Dietrich G, Vergnolle N, Altier C (2015). TRPV1 sensitization mediates postinflammatory visceral pain following acute colitis. Am J Physiol Gastrointest Liver Physiol.

[CR15] Longo V, Tamma R, Brunetti O, Pisconti S, Argentiero A, Silvestris N (2018) Mast cells and angiogenesis in pancreatic ductal adenocarcinoma. 18:319–323. doi:10.1007/s10238-018-0493-6 10.1007/s10238-018-0493-6 29492715

[CR16] Matricon J, Muller E, Accarie A, Meleine M, Etienne M, Voilley N, Busserolles J, Eschalier A, Lazdunski M, Bourdu S, Gelot A, Ardid D (2013). Peripheral contribution of NGF and ASIC1a to colonic hypersensitivity in a rat model of irritable bowel syndrome. Neurogastroenterol Motil.

[CR17] Miao XR, Fan LC, Wu S, Mao QX, Li Z, Lutz BM, Xu J, Lu ZJ, Tao YX (2017). DNMT3a contributes to the development and maintenance of bone cancer pain by silencing Kv1.2 expression in spinal cord dorsal horn. Mol Pain.

[CR18] Nigri J (2017). PAP/REG3A favors perineural invasion in pancreatic adenocarcinoma and serves as a prognostic marker. Cell Mol Life Sci.

[CR19] Prato V, Taberner FJ, Hockley JRF, Callejo G, Arcourt A, Tazir B, Hammer L, Schad P, Heppenstall PA, Smith ES, Lechner SG (2017). Functional and molecular characterization of mechanoinsensitive “Silent” nociceptors. Cell Rep.

[CR20] Quartu M, Carozzi VA, Dorsey SG, Serra MP, Poddighe L, Picci C, Boi M, Melis T, del Fiacco M, Meregalli C, Chiorazzi A, Renn CL, Cavaletti G, Marmiroli P (2014). Bortezomib treatment produces nocifensive behavior and changes in the expression of TRPV1, CGRP, and substance P in the rat DRG, spinal cord, and sciatic nerve. Biomed Res Int.

[CR21] Stopczynski RE, Normolle DP, Hartman DJ, Ying H, DeBerry JJ, Bielefeldt K, Rhim AD, DePinho RA, Albers KM, Davis BM (2014). Neuroplastic changes occur early in the development of pancreatic ductal adenocarcinoma. Cancer Res.

[CR22] Theoharides TC (2008). Mast cells and pancreatic cancer. N Engl J Med.

[CR23] Wang GD, Wang XY, Liu S, Qu M, Xia Y, Needleman BJ, Mikami DJ, Wood JD (2014). Innervation of enteric mast cells by primary spinal afferents in guinea pig and human small intestine. Am J Physiol Gastrointest Liver Physiol.

[CR24] Wang L, Xu H, Ge Y, Zhu H, Yu D, Yu W, Lu Z (2017). Establishment of a murine pancreatic cancer pain model and microarray analysis of painassociated genes in the spinal cord dorsal horn. Mol Med Rep.

[CR25] Xu XJ, Zhang YL, Liu L, Pan L, Yao SK (2017). Increased expression of nerve growth factor correlates with visceral hypersensitivity and impaired gut barrier function in diarrhoea-predominant irritable bowel syndrome: a preliminary explorative study. Aliment Pharmacol Ther.

[CR26] Zhang L, Song J, Bai T, Wang R, Hou X (2018). Sustained pain hypersensitivity in the stressed colon: role of mast cell-derived nerve growth factor-mediated enteric synaptic plasticity. Neurogastroenterol Motil.

[CR27] Zhang W, Gao J, Zhao T, Wei L, Wu W, Bai Y, Zou D, Li Z (2011). Proteinase-activated receptor 2 mediates thermal hyperalgesia and is upregulated in a rat model of chronic pancreatitis. Pancreas..

[CR28] Zhu Y, Colak T, Shenoy M, Liu L, Pai R, Li C, Mehta K, Pasricha PJ (2011). Nerve growth factor modulates TRPV1 expression and function and mediates pain in chronic pancreatitis. Gastroenterology..

