# Supplementary material for: Inhibition of Mast Cell Degranulation Relieves Visceral Hypersensitivity Induced by Pancreatic Carcinoma in Mice
Source: J Mol Neurosci. 2019 Jun 14;69(2):235–45. doi: 10.1007/s12031-019-01352-6 (PMC6732154; doi:10.1007/s12031-019-01352-6)
Supplement: Supplementary file 1 — (DOCX 17 kb) [file 12031_2019_1352_MOESM1_ESM.docx]

**Supplemental table**

**Table 1 Clinical Data of Human Pancreatic Carcinoma Specimens**

| **No.** | **Sex** | **Age** | **Tumor size**  **(cm×cm)** | **Pathology** | **VAS** |
| --- | --- | --- | --- | --- | --- |
| 1 | M | 57 | 3×2.4 | Pancreatic head ductal adenocarcinoma | 5 |
| 2 | M | 48 | 2.5×2 | Pancreatic head ductal adenocarcinoma | 5 |
| 3 | M | 55 | 3×2.4 | Pancreatic head ductal adenocarcinoma | 5 |
| 4 | F | 60 | 4×3 | Pancreatic head ductal adenocarcinoma | 7 |
| 5 | F | 58 | 2.8×2.5 | Pancreatic head ductal adenocarcinoma | 8 |
| 6 | M | 61 | 2.6×2.4 | Pancreatic head ductal adenocarcinoma | 8 |
| 7 | M | 53 | 4×3.5 | Pancreatic body ductal adenocarcinoma | 5 |
| 8 | F | 51 | 4.5×3.5 | Pancreatic body ductal adenocarcinoma | 6 |
| 9 | M | 57 | 3×2.6 | Pancreatic body ductal adenocarcinoma | 6 |

**Table 2 Clinical Data of Human Normal Pancreas Specimens**

| **No.** | **Sex** | **Age** | **Surgery** | **Pathology** | **VAS** |
| --- | --- | --- | --- | --- | --- |
| 1 | M | 62 | Pancreaticoduodenectomy | Distal bile duct carcinoma | 0 |
| 2 | M | 51 | Pancreaticoduodenectomy | Distal bile duct carcinoma | 0 |
| 3 | M | 50 | Pancreaticoduodenectomy | Duodenal papillary carcinoma | 0 |
| 4 | F | 48 | Pancreaticoduodenectomy | Distal bile duct carcinoma | 0 |
| 5 | M | 51 | Pancreaticoduodenectomy | Distal bile duct carcinoma | 0 |
| 6 | F | 46 | Pancreaticoduodenectomy | Distal bile duct carcinoma | 0 |
| 7 | M | 26 | Liver donation | Normal pancreas | 0 |
| 8 | M | 28 | Liver donation | Normal pancreas | 0 |
| 9 | M | 36 | Liver donation | Normal pancreas | 0 |
